# Fatty acid binding protein 7 may be a marker and therapeutic targets in clear cell renal cell carcinoma

**DOI:** 10.1186/s12885-018-5060-8

**Published:** 2018-11-15

**Authors:** Kazuhiro Nagao, Nachi Shinohara, Frank Smit, Mirjam de Weijert, Sander Jannink, Yuji Owada, Peter Mulders, Egbert Oosterwijk, Hideyasu Matsuyama

**Affiliations:** 1 Department of Urology, Graduate School of Medicine, Yamaguchi University, 1-1-1, Minami-Kogushi, Ube, Yamaguchi, 755-8505 Japan; 2 Department of Urology, Radboud University Medical Center, Nijmegen 267 Experimental Urology, Geert Grooteplein, 26-28, P.O. Box 9101, NL-6525 GA Nijmegen, The Netherlands; 3 Department of Organ Anatomy, Tohoku University Graduate School of Medicine, 2-1, Seiryo-machi, Aoba-ku, Sendai, 980-8575 Japan

**Keywords:** Fatty acid binding protein 7, Clear cell renal cell carcinoma, Prognostic marker, Therapeutic target, Fatty acid binding protein 6

## Abstract

**Background:**

To identify potential therapeutic target in clear cell renal cell carcinoma (ccRCC), we performed a transcriptome analysis. Our analysis showed that fatty acid binding protein 7 (FABP7) has the highest mean differential overexpression in ccRCC compared to normal kidney. We aimed to investigate the significance of FABP7 in ccRCC.

**Methods:**

Immunohistochemical staining for 40 advanced ccRCC cases was performed to investigate correlation between clinicopathological parameters and FABP7. They were composed of 40–83 years old cases with 33 male, 22 cases with pT ≥ 3, 19 cases with M1, and 16 cases with grade 3. The effect of gene knockdown was analysed by a cell viability assay and invasion assay in FABP7-overexpressing cell lines (SKRC7 and SKRC10).

**Results:**

Our immunohistochemical analysis showed that higher FABP7 expression significantly correlated with distant metastasis and poor cancer-specific survival (CSS; both *p* < 0.05). Functional suppression of FABP7 significantly inhibited SKRC10 cell growth (p < 0.05) and resulted in a significant reduction of the invasive potential (*p* < 0.01), but did not cause growth inhibition of SKRC7 cells. We found that The Cancer Genome Atlas Research Network (TCGA) database shows FABP6 and 7 as equally overexpressed in the FABP family. Functional suppression of fatty acid binding protein 6 (FABP6) resulted in significant growth inhibition of SKRC7 cells (*p* < 0.005).

**Conclusions:**

Functional suppression of FABP7 significantly reduced cell viability and invasive potential in a ccRCC cell line. FABP7 may play a role in progression in some metastatic ccRCCs. The suppressed function may be compensated by another FABP family member.

## Background

Renal cell carcinoma (RCC) accounts for 3% of all cancer cases in adults, and it is the 11th most common cancer in men and the 14th most common in women. Its worldwide incidence and associated mortality in 2012 were ~ 337,000 new cases and 143,000 deaths [1], both of which have increased steadily over the years [2, 3].

Clear cell RCC (ccRCC) is the most prevalent pathological subtype of this cancer, accounting for 80% of all cases. Recent advances in our understanding of the underlying genetic events leading to ccRCC have given rise to treatment modalities targeting the vascular endothelial growth factor (VEGF) signaling axis and its related pathways. Although VEGF tyrosine kinase inhibitors (VEGF-TKIs) and mammalian target of rapamycin (mTOR) inhibitors significantly extend the progression free survival of patients with metastatic RCC [4], drug resistance invariably occurs.

Recently, cabozantinib, an inhibitor of tyrosine kinases including MET, VEGF receptor, and Anexelekto [5], and nivolumab, an immune check point inhibitor targeting programmed cell death 1 on tumor-infiltrating lymphocytes [6], became available for clinical use. But it is still necessary to identify new molecular targets for the treatment of patients with metastatic ccRCC to improve the treatment outcome. To identify new therapeutic targets, we performed a transcriptome analysis of ccRCC and normal kidney samples. Among the up-regulated genes in ccRCC, we focused on the five most differentially expressed genes; prolyl hydroxylase 3 (PHD3), fatty acid binding protein 7 (FABP7), carbonic anhydrase IX (CAIX), NADH dehydrogenase 1 alpha subcomplex, 4-like 2 (NDUFA4L2), and monocarboxylate transporter 4 (MCT4).

FABP7 is known to be upregulated in brain tissue, and its function is well studied in glioblastoma cell lines. Zhou et al. reported that FABP7 is significantly up-regulated in ccRCC and that the expression of FABP7 positively correlates with advanced clinical stage and poor survival of patients with ccRCC [7]. Overexpression of FABP7 in RCC cells enhances cell growth and cell cycle progression, which activates both extracellular-signal-regulated kinases (ERK) and signal transducer and activator of transcription 3 (Stat3) signaling.

To the best of our knowledge, this is the first report about the complementary role of fatty acid binding protein 6 (FABP6) for FABP7 in ccRCC.

## Methods

### Transcriptome analysis

Total RNA was extracted from 60 ccRCC samples (49 cases without metastasis, and 11 cases with synchronous metastasis at nephrectomy), and 20 corresponding normal kidney samples using the TRIzol Reagent (Invitrogen®). The tumor samples contained > 70% tumor cells as estimated by microscopy. Gene expression analysis was performed on the Genechip Human Gene 1.0 ST Array (Affymetrix®), according to the manufacturers’ instructions. The expression profiles were analyzed using an unsupervised hierarchical average linkage-clustering algorithm. The analysis was ethically approved at our institution (Radboud University Medical Center, Nijmegen).

### Antibodies

The following primary antibodies were used: anti-CAIX monoclonal antibody M75 (HB-11128, ATCC), rabbit anti-NDUFA4L2 (Proteintech®), anti-PHD3 monoclonal antibody (Novus Biologicals®), rabbit anti-MCT4 (Millipore®), rabbit anti-FABP7 polyclonal antibody (produced by a co-author, YO [8]), and a mouse anti-Stat3 monoclonal antibody (Abcam®). Primary antibodies were detected using a horseradish peroxidase (HRP)-conjugated rabbit anti-mouse immunoglobulin antibody) (DAKO®) and HRP-conjugated swine anti-rabbit immunoglobulin antibody (DAKO®) (secondary antibodies).

### Immunohistochemistry

For immunohsitochemical analysis, we used the slides of paraffin-embedded ccRCC and normal kidney samples extracted from the same cases used in our transcriptome analysis.

Slides of paraffin-embedded ccRCC samples were deparaffinized in xylene, followed by incubation in a graded series of ethanol (100, 70 and 50%) and re-hydration in PBS. The slides were immersed in 3% H_2_O_2_ for 30 min at room temperature to block endogenous peroxidase activity. After a PBS wash, the slides were immersed in 10 mM citrate buffer (pH 6.0) and heated in a microwave oven for 10 min at 100 °C. After cooling to room temperature, the slides were blocked in 20% normal goat serum for 10 min. The tissue slices were then incubated with the mouse anti-CAIX monoclonal M75 antibody (1600 dilution), rabbit anti-NDUFA4L2 polyclonal antibody (12,000 dilution), mouse anti-PHD3 monoclonal antibody (1200 dilution), rabbit anti-MCT4 polyclonal antibody (1200), or the rabbit anti-FABP7 polyclonal antibody (11000) for 90 min at room temperature, washed in PBS, and then incubated with the appropriate secondary antibody for 1 h at room temperature (1100 dilution). After washing with PBS, the slides were incubated in a PowerVision DAB solution (40 μl of Solution A + 40 μl of Solution B + 5 μl of Tween 20 brought up to 1 ml with PBS, ImmunoLogic®) for 10 min at room temperature, washed again in tap water, counterstained with hematoxylin, and mounted in Permount (Fisher Scientific®).

### Patients’ characteristics in the cases analyzed by FABP7 immunohistochemical staining

We retrospectively reviewed the clinical data of 40 patients with metastatic ccRCC who had undergone nephrectomy, followed by systemic therapy using either a cytokine, VEGF-TKIs, or mTOR inhibitors in Yamaguchi University Hospital. The analysis was ethically approved at our institution (Yamaguchi University Hospital).

### Evaluation of FABP7 expression status in ccRCC specimens

We scored the FABP7 staining index based on the staining intensity (Fig. 2a, low: 1, intermediate: 2, high: 3, and nuclear staining: 4 points) and the number of the cells as calculated by the formula: (staining intensity 1, 2, 3, or 4) × cell counts per 500 cells in each sample of ccRCC specimen. The mean value of the index was 11 in all the cases analyzed. We defined the case with more than mean value of the index as high FABP7 expression case, and also defined the case with less than mean value of the index as low FABP7 expression case.

### RNA extraction and real-time quantitative RT-PCR

Total RNA was extracted from the cultured cells using the TRIzol Reagent (Invitrogen®). RNA was used for reverse transcription with SuperScript II RNase Hˉ Reverse Transcriptase (Invitrogen®). The cDNA synthesis was performed using 2 μg of RNA with a mix of reverse transcriptase, 5× first-strand buffer, DTT, dNTPs, and random hexamers.

qRT-PCR was run using a LightCycler 480 Real-Time PCR System (Roche®). The SYBR Green method was used to measure *hypoxanthine phosphoribosyltransferase 1* (*HPRT*), *CAIX*, *MCT4*, *FABP7* and *FABP6* mRNA expression and the TaqMan method to measure *HPRT*, *PHD3*-, and *NDUFA4L2* mRNA expression levels. The primer sequences were as follows:

- HPRT
  Forward primer sequence: 5’CTCAACTTTAACTGGAAAGAATGTC3’
  Reverse primer sequence: 5’TCCTTTTCACCAGCAAGCT3’
  Probe sequence: 5’TTGCTTTCCTTGGTCAGGCAGTATAATC3’
- *CAIX*
  Forward primer sequence: 5’TAAGCAGCTCCACACCCTCT3’
  Reverse primer sequence: 5’TCTCATCTGCACAAGGAACG3’
- *MCT4*
  Forward primer sequence: 5’GCACCCACAAGT TCTCCAGT3’
  Reverse primer sequence: 5’CAAAATCAGGGAGGAGGTGA3’
- *PHD3*
  Forward primer sequence: 5’GAATTGGGATGCCAAGCTACA3’
  Reverse primer sequence: 5’TGACCAGAAGAACAGGAGTCTGTC3’
  Probe sequence: 5’ATGGGCTCCACATCTGCTATGAATGATTTC3’
- *NDUFA4L2*
  Forward primer sequence: 5’GACGTCTGCTGGGACAGAAAG3’
  Reverse primer sequence: 5’AGTGGAAACTGCAAGGAACTTGTA3’
  Probe sequence: 5’CCGGAGCCCTGGAACCGC3’
- *FABP7*
  Forward primer sequence: 5’CTCAGCACATTCAAGAACACG3’
  Reverse primer sequence: 5’CCATCCAGGCTAACAACAGAC3’
- *FABP6*
  Forward primer sequence: 5’ACTACTCCGGGGGCCACACCAT3’
  Reverse primer sequence: 5’GTCTCTTGCTCACGCGCTCATAGG3’

*HPRT* mRNA expression served as an internal control. All the measurements were repeated at least twice to confirm reproducibility. The expression of the target mRNA was quantified relative to that of the *HPRT* mRNA and untreated controls were used as a reference according to the model described by Pfaffl [9].

### Cell culture

Human ccRCC-derived cell lines (SKRC1, SKRC7, SKRC10, SKRC12, SKRC17, SKRC59, and CaKi1) were cultured in the RPMI 1640 medium supplemented with 10% of fetal calf serum and ʟ-glutamate (Gibco®) in a humidified atmosphere containing 5% of CO_2_ at 37 °C.

### Western blot analysis

Protein expression levels were determined by western blot analysis. In brief, cells were lysed in a buffer consisting of 20 mM Tris-HCl (pH 7.5), 150 mmol/l NaCl, 0.1% SDS, 5 mmol/l EDTA, 1% of Triton X-100 (Sigma-Aldrich®), and 1 tablet of a protease inhibitor cocktail (cOmplete Mini, Roche®) per 10 mL of the buffer. The lysates were centrifuged at 13,200 rpm for 15 min at 4 °C, and the supernatants were collected to determine protein concentration using the BSA protein assay reagent. Total protein (10 or 20 μg) was separated by SDS-PAGE in a 10% gel and transferred to a filter using a semidry blotter. After blocking with 1% skimmed milk powder dissolved in PBS, the blots were incubated with the appropriate primary and secondary antibodies: the rabbit anti-FABP7 polyclonal antibody (1,5000) and mouse anti-Stat3 monoclonal antibody (110000). Detection was performed using the Amersham ECL Plus Western Blotting Detection Reagents kit (Amersham®), and protein expression levels were quantified in the ImageJ software (NIH, USA).

### The knockdown with small interfering RNA (siRNA)

A commercially available mixture of 4 single-stranded 19-bp siRNAs (ON-TARGETplus SMARTpool, Invitrogen®) was used to transfect SKRC7 and SKRC10 cells according to the manufacturer’s instructions. The sequences of the siRNAs were 5’CAACGGUAAUUAUCAGUCA3’, 5’GUCAGAACUUUGAUGAGUA3’, 5’GAACACGGAGAUUAGUUUC3’, and 5’GAUGAUAGAAACUGUAAGU3’ for *FABP7*, 5’UCGGAGGCGUGACCUAUGA3’, 5’CCUCAGAGAUCGUGGGUGA3’, 5’GUGAGAAGAAUUAUGAUGA3’, and 5’GCAAGGAAAGCAACAUACA3’ for *FABP6*.

The final concentration of siRNA for transfection was 33 nmol/L. A scrambled siRNA served as a negative control (Invitrogen®). Transfection was carried out using Lipofectamine 2000 (Invitrogen®).

### Cell viability assay

Cell viability was tested by a 3-(4,5-dimethylthiazol-2-yl)-2,5-diphenyltetrazolium bromide (MTT) assay.

SKRC7 and SKRC10 cells transfected with scrambled siRNA, *FABP7* siRNA, or *FABP6* siRNA were seeded in triplicate in 96-well plates at 4000 cells per well and cultured at 37 °C and 5% CO_2_ for up to 7 days. On the day of measurement, 30 μL of MTT Thiazolyl Blue (5 mg/mL: Sigma-Aldrich®) was added into each well, and the cells were incubated for an additional 4 h. Subsequently, 100 μL of DMSO was added, and the plate was shaken for 5 min at room temperature to dissolve the formazan crystals. Finally, optical density (OD) at 595 nm was measured (3550 Microplate Reader, Bio-rad®).

### Cell invasion assay

This assay was conducted using a BD BioCoat Matrigel Invasion Chamber (BD-Biosciences®).

SKRC10 cells were harvested 48 h after transfection at 37 °C. The transfected cells were re-suspended in serum-free Dulbecco’s modified Eagle’s medium and then added to the upper chamber at a density of 2 × 10^5^ cells/well. After 24 h of incubation at 37 °C, cells migrating through the membrane were stained. The results are expressed as invading cells quantified at OD 560 nm.

### Statistical analysis

Student’s *t* test was used for statistical analysis using commercially available software (JMP version 4, SAS). A different with a *p*-value less than 0.05 was considered significant.

## Results

### Transcriptome analysis

**Fig. 1:**
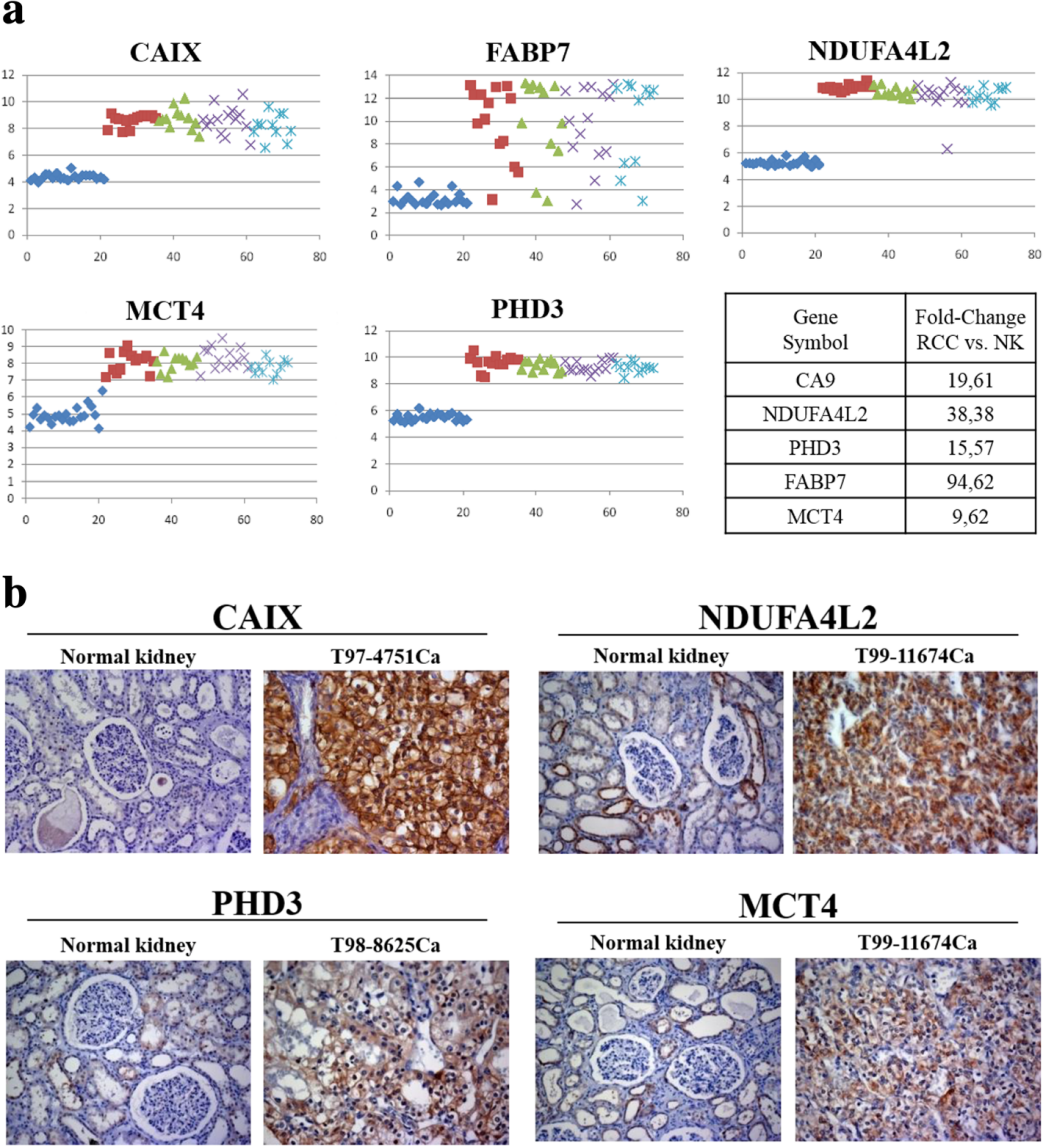
Highly overexpressed molecules in ccRCC samples. **a** Highly upregulated genes based on the transcriptome analysis. The figure shows fold- changes of differential expression for highly upregulated genes. ●: normal kidney, ■: RCC that never metastasized, ▴: RCC that metastasized after nephrectomy, ×: RCC with metastasis prior to nephrectomy, ✳: metastatic RCC.Vertical and horizontal axes indicate fold changes in mRNA expression and numbers of cases, respectively. **b** Immunohistochemical staining for PHD3, CAIX, NDUFA4L2, and MCT4. Representative cases of immunohistochemical staining in paraffin-imbedded tissues slices (magnification: × 400)

This analysis revealed numerous genes that were over-expressed in ccRCC samples compared to normal kidney tissues (Table 1). Five genes (*PHD3*, *FABP7*, *CAIX*, *NDUFA4L2*, and *MCT4*) were expressed approximately 10-fold higher in ccRCC specimens compared to normal kidney samples (Fig. 1a). Higher expression levels of these genes were also observed in The Cancer Genome Atlas Research Network (TCGA) database. The mRNA expression levels of *PHD3*, *CAIX*, *NDUFA4L2*, and *MCT4* were equally high in most ccRCC samples, and did not depend on cancer progression. In contrast, the levels of *FABP7* mRNA varied. Among the overexpressed genes, *FABP7* had the highest mean expression level at the mRNA level, and the expression levels varied depending on the ccRCC cases.

**Table 1:** Highly mRNA overexpression in ccRCC compared to normal kideny

Gene Symbol	Ref Seq	LSMean (NK)	LSMean (RCC)	Fold-Change (RCC/NK)
FABP7	NM_001446	3.20163	9.76575	94.6231
NDUFA4L2	NM_020142	5.26123	10.5235	38.3803
CP	NM_000096	5.94544	10.8426	29.7987
HIG2	NM_013332	4.40522	9.16447	27.0818
ANGPTL4	NM_139314	3.92434	8.28295	20.5151
CA9	NM_001216	4.36817	8.66135	19.6054
ANGPT2	NM_001147	4.81109	8.97052	17.8695
EGLN3	NM_022073	5.49587	9.45616	15.5656
C3	NM_000064	7.32719	11.2612	15.2848
AHNAK2	NM_138420	3.73036	7.61734	14.7944
NETO2	NM_018092	4.68377	8.51052	14.1895
ENO2	NM_001975	4.74515	8.47351	13.254
NNMT	NM_006169	5.63127	9.29046	12.6335
LOX	NM_002317	6.23912	9.89302	12.5873
CYP2J2	NM_000775	4.48354	8.08306	12.1217
TNFAIP6	NM_007115	3.21484	6.78895	11.9101
PNMA2	NM_007257	4.42318	7.86148	10.8401
RRM2	NM_001034	3.95228	7.36327	10.6368
VWF	NM_000552	5.54317	8.90743	10.2978
CXCL10	NM_001565	4.73448	8.09726	10.2872
NPTX2	NM_002523	4.07029	7.42967	10.263
TMEM45A	NM_018004	3.75004	7.07615	10.029
CXCL11	NM_005409	3.39841	6.6673	9.63905
SLC16A3	NM_001042422	4.92069	8.18727	9.62364
IGFBP3	NM_001013398	7.18489	10.4391	9.54115
ENPP3	NM_005021	6.77557	9.96963	9.15181
DNAH11	NM_003777	3.34526	6.53492	9.12395
SCD	NM_005063	6.71436	9.89784	9.08496
CXCL9	NM_002416	4.06238	7.23311	9.00504
FCGR3A	NM_000569	4.98514	8.13677	8.88659
BHLHE41	NM_030762	5.29995	8.40999	8.63406
HK2	NM_000189	4.30467	7.39848	8.53744
PCSK6	NM_002570	4.33254	7.34167	8.05077
GBP5	NM_052942	4.17666	7.1851	8.04697
ESM1	NM_007036	6.14498	9.1468	8.0101
PDK1	NM_002610	6.19808	9.19637	7.99055

### Immunohistochemistry

Immunohistochemical staining was used to determine whether the differential mRNA expression of these genes was reflected in differential protein expression in the ccRCC and normal kidney samples. Homogenous staining was observed in ccRCC samples for PHD3, CAIX, NDUFA4L2, and MCT4, while the staining level of FABP7 varied among the cases. Representative cases of differential immunohistochemical staining for PHD3, CAIX, NDUFA4L2, and MCT4 in ccRCC and normal kidney samples are shown in Fig. 1b. CAIX was not expressed in normal kidneys. PHD3 expression was weak in all epithelial cells of the kidney, whereas MCT4 and NDUFA4L2 expression was restricted to the distal tubuli.

### FABP7 immunohistochemical staining as a prognostic marker

We retrospectively reviewed the clinical data of 40 patients with metastatic ccRCC who had undergone nephrectomy, followed by systemic therapy using either a cytokine, VEGF-TKIs, or mTOR inhibitors in Yamaguchi University Hospital. The analysis was ethically approved at our institution (Yamaguchi University Hospital).

**Fig. 2:**
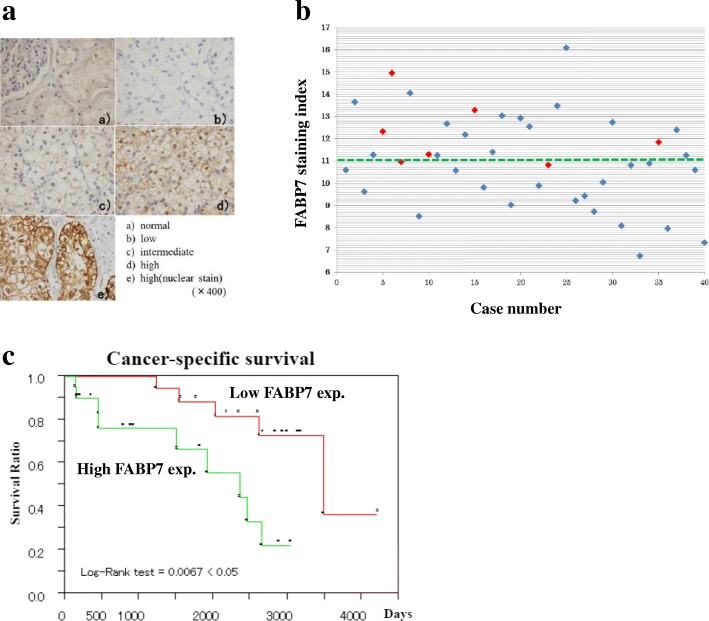
FABP7 expression in ccRCC samples. **a** Immunohistochemical staining for FABP7. Different immunohistochemical staining intensities in paraffin-imbedded tissues slices (magnification: × 400) are shown. Vertical axis shows the FABP7 staining index. Eleven is the mean value of the index in 40 cases. The horizontal axis shows the number of cases. **b** Distribution of FABP7 expression in clinical cases. Vertical axes show the FABP7 staining index. Eleven is the mean value of the index in 40 cases. The horizontal axis shows the number of the cases. ●: Male case, ●: Female case. **c** Correlation between FABP7 expression and cancer-specific survival. Vertical axis shows the cancer-specific survival rate. The horizontal axis shows the days after treatment

To evaluate the efficacy of FABP7 as a biomarker, we retrospectively reviewed the clinical data of 40 patients with metastatic ccRCC who had undergone nephrectomy, followed by systemic therapy using either a cytokine, VEGF-TKIs, or mTOR inhibitors in Yamaguchi University Hospital. The expression pattern of FABP7 was consistent in the cytoplasm, while nuclear expression varied. The expression of FABP7 was frequently observed in the peripheral lesion of the tumor with viable cancer cell, while was not in the central lesion with necrotic tissue. The expression level of FABP7 depended on the cases in agreement with transcriptome analysis. Distribution status of the FABP7 staining index among the 40 ccRCC cases is shown in Fig. 2b. We classified the cases into those with higher or lower expression of FABP7 based on the FABP7 staining index.

Table 2 depicts the relation of patient characteristics to FABP7 expression. The cases with higher FABP7 expression significantly correlated with distant metastasis and corrected calcium level (both, *p* < 0.05). Then we analysed the role of FABP7 expression as a prognostic marker. In our cohort, other known prognostic factors (pT stage, grade, Hb, neutrophil, LDH, CRP, Na, corrected calcium) could not show significant correlation with cancer-specific survival (CSS), while FABP7 could be a prognostic marker (Fig. 2c). Cases with higher FABP7 expression significantly correlated with poor CSS as compared to those with lower expression (*p* < 0.05).

**Table 2:** Correlation between clinicopathological characteristics and FABP7 expression

		FABP7 index		
Characteristics	Criteria	High (> 11)	High (< 11)	*P* value
Age (years)	High (≥65)	9	12	0.5273
	Low (< 65)	11	8	
Gender	Male	15	18	0.4075
	Female	5	2	
Grade	High (3)	10	6	0.3332
	Low (1 or 2)	10	13	
pT stage	High (3)	11	11	1
	Low (1 or 2)	8	9	
N stage	+	1	0	1
	–	18	19	
M stage	+	13	6	< 0.05
	–	4	11	
Natrium (mEq/l)	High (> 142)	1	4	0.3398
	Low (≤142)	18	15	
Albumin (g/dl)	High (≥3.7)	8	12	0.5027
	Low (< 3.7)	9	7	
Corrected Calcium (mg/dl)	High (≥10)	9	3	< 0.05
	Low (< 10)	8	15	

### Functional effects of a *FABP7* knockdown

**Fig. 3:**
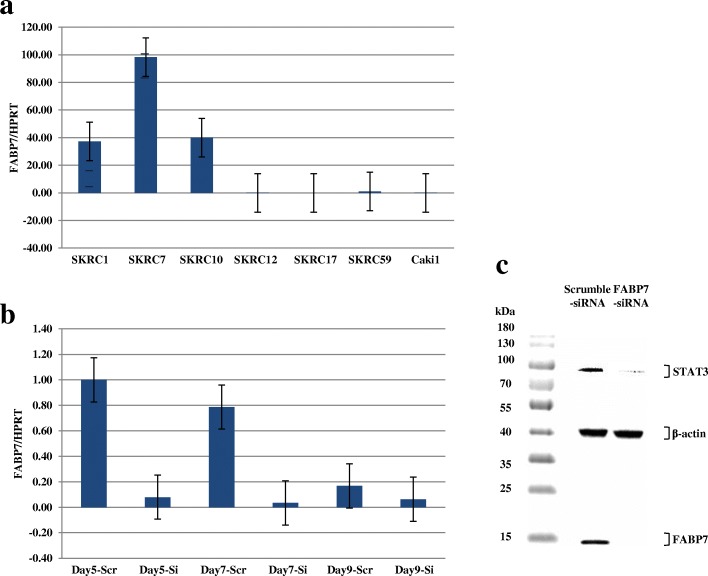
FABP7 expression in ccRCC cell lines. **a** Relative mRNA expression of *FABP7* in ccRCC cell lines. Relative mRNA expression levels in ccRCC cell lines are shown. Vertical bars indicate the ratio of crossing point (CP) values (*FABP7*/*HPRT*). **b** Relative mRNA expression levels after a knockdown of *FABP7*. Relative mRNA expression levels after *FABP7*- siRNA transfection of SKRC10 cells are shown. Vertical bars indicate the ratio of crossing point (CP) values (*FABP7*/*HPRT*). **c** FABP7 protein expression after the *FABP7* knockdown. Expression of FABP7 and Stat3 (molecular weights 15 and 88 kDa, respectively) decreased after *FABP7*- siRNA transfection into SKRC10 cells

The mRNA and protein expression levels of FABP7 were evaluated in 7 ccRCC-derived cell lines: SKRC1, SKRC7, SKRC10, SKRC12, SKRC17, SKRC59, and Caki1. The mRNA levels are shown in Fig. 3a. SKRC1, SKRC7, and SKRC10 showed higher expression levels of *FABP7* compared to the other cell lines.

We transfected *FABP7*- siRNA into SKRC10 cells to knock down the expression of *FABP7*. This transfection resulted in a > 90% reduction of mRNA expression for 7 days as compared to scramble transfection (Fig. 3b), and the protein level was also reduced (Fig. 3c). Interestingly, FABP7 knockdown reduced the expression of Stat3 (Fig. 3c).

**Fig. 4:**
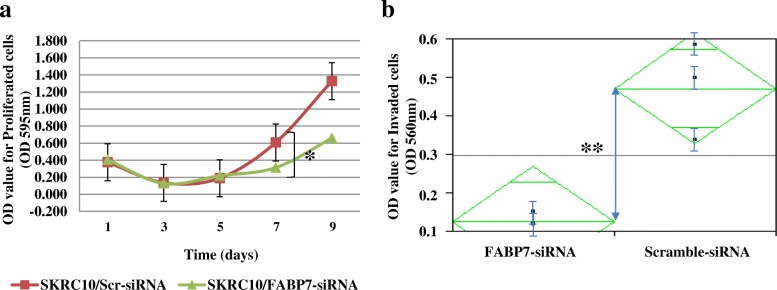
Effect of *FABP7* knockdown in a ccRCC cell line. **a** The cell viability assay after the *FABP7* knockdown. Proliferating SKRC10 cells after the knockdown of *FABP7* expression were assessed by an MTT assay. Vertical bars indicate the value of optical density (OD) of the cells at 595 nm. *p < 0.05. **b** Invasion assay after the *FABP7* knockdown. Invading SKRC10 cells after the knockdown of *FABP7* were analyzed by the Matrigel Invasion assay. Vertical axis indicates the value of optical density (OD) of the cells at 560 nm. **p < 0.01

We evaluated the effect of the *FABP7* knockdown on SKRC10 cells by the MTT assay. Functional suppression of *FABP7* resulted in significant growth inhibition of SKRC10 cells at 7 days after transfection (*p* < 0.05, Fig. 4a). A Matrigel invasion assay indicated a significant reduction in the invasive potential by the functional suppression of *FABP7* (*p* < 0.01, Fig. 4b).

### *FABP6* expression

**Fig. 5:**
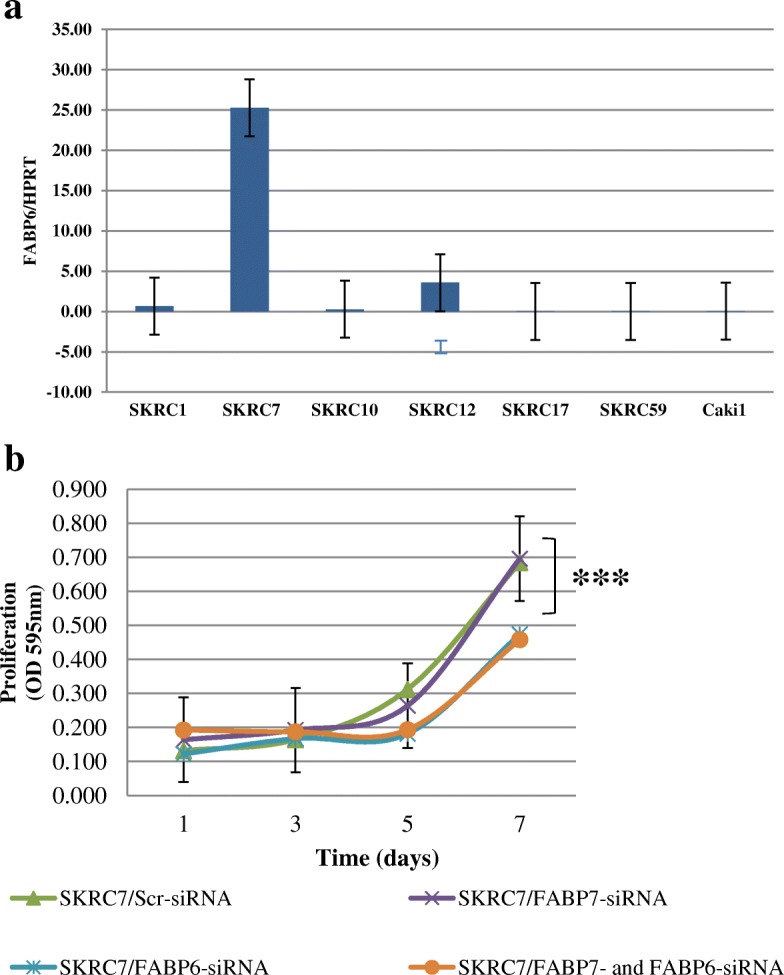
Effect of *FABP7* knockdown in ccRCC cell lines. **a** Relative mRNA expression of FABP6 in ccRCC cell lines. Relative mRNA expression levels in ccRCC cell lines are shown. Vertical axis indicates the ratio of C*P* values (*FABP6*/*HPRT*). **b** Cell viability assay after FABP7 or/and FABP6 knockdown. Proliferating SKRC7 cells after the knockdown of *FABP7* or/and *FABP6* were analyzed by the MTT assay. Vertical axis indicates the value of optical density (OD) of the cells at 560 nm. **p < 0.05

Although higher expression of *FABP7* was observed in SKRC7 cells than in SKRC10 cells, suppression of *FABP7* in SKRC7 cells did not affect the cell viability as shown in Fig. 5b. Therefore, we analysed relative mRNA expression of *FABP6*, a member of the FABP family, in ccRCC cell lines and showed high expression of *FABP6* mRNA in SKRC7 cells, while SKRC10 cells had low expression of *FABP6* mRNA (Fig. 5a).

We evaluated the effect of the *FABP6* knockdown on SKRC7 cells by the MTT assay. Functional suppression of *FABP6* resulted in significant growth inhibition of SKRC10 cells at 7 days after transfection (*p* < 0.05, Fig. 5b), while combined knock down of FABP7 and FABP6 did not inhibit the cell growth more than knock down of FABP6 alone. In SKRC7 cells, FABP6 seemed to compensate the role of FABP7 in cell proliferation.

## Discussion

Most cancer cells undergo a metabolic shift from electron transport chain to glycolysis in producing energy, which resulted in synthesizing proteins and lipids. Increased glucose uptake and the accumulation of lactate are common features of cancer cells even under normoxic conditions named as the Warburg effect [10, 11]. HIF-1α promotes angiogenesis by inducing VEGF [12], which upregulates the transcription of various glycolytic genes to mediate a metabolic shift that shunts glucose metabolites away from mitochondria [13]. In ccRCC, hypoxia-inducible factor- 1α (HIF-1α) is overexpressed due to functional loss of the von Hippel-Lindau (VHL) protein. Recent studies showed that the Warburg Effect in cancer cells can be targeted by inhibition of glucose transporter 1, [14], or conversion of lactate to pyruvate by lactate dehydrogenase A (LDH-A) [15]. HIF-1a was reported to regulate the expression of fatty acid synthase (FASN) [16], which facilitate fatty acid synthesis and lipid storage [17]. Recently, several FASN inhibitors were reported to show antitumor effect against breast [18], ovarian [19], and prostate cancers [20] in preclinical models.

Based on our transcriptome analysis, we focused on the five most differentially expressed genes: *PHD3*, *FABP7*, *CAIX*, *NDUFA4L2*, and *MCT4*. All the genes have been regarded as working downstream of HIF in relation to hypoxia, metabolism, and pH regulation in cancer, which are supposed to induce the Warburg effect [21]. Our results are in good agreement with a meta-analysis of 5 ccRCC expression datasets available in Oncomine [22], in which *NDUFA4L2*, *PHD3*, *CAIX*, and *MCT4* showed the 1st, 2nd, 7th, and 11th highest expression level, respectively. In addition, our data indicate that these genes are equally highly expressed in tumor samples of non-metastatic and metastatic cases, which revealed that these genes are stably over-expressed in the vast majority of ccRCCs compared to normal kidney tissue regardless of the metastatic potential or tumor stage. In contrast, heterogeneous distribution of mRNA expression of *FABP7* with a higher average value leads to the hypothesis that FABP7 may be involved in cancer progression in ccRCC.

FABP7 is a member of the FABP family, which is known to have family members 1 to 7 in cancer tissue. Bensaad et al. showed that a knockdown of FABP3, FABP7, or Adipophilin impaired lipid droplet formation under hypoxic conditions in glioblastoma and breast cancer cell lines [23]. They supposed that inhibition of lipid storage may reduce the ability in protecting the cancer cells from toxicity by reactive oxygen and decrease the survival of the cancer cells under the condition of hypoxia-reoxygenation. Liu et al. reported that FABP7 is significantly up-regulated in triple-negative breast cancer and that the high expression level of FABP7 is associated with poor prognosis [24]. They also showed that depletion of FABP7 significantly reduced the growth rate of cancer cells, which leads to sensitize the growth inhibition by omega-3 docosahexaenoic acid (DHA). We also added DHA to the *FABP7*- knockdown SKRC10 cells in a cell viability assay, but growth inhibition was not affected (data not shown). In FABP7-overexpressing RCC cells, Zou et al. showed that overexpression of FABP7 in RCC cells promotes cell growth by the activation of ERK and Stat3 signaling pathways [7]. In our study, it was also confirmed that the knockdown of FABP7 reduced Stat3 expression, which may result in growth inhibition and reduction of the invasive potential.

On the other hand, Takaoka et al. reported that the levels of FABP7 expressed in a ccRCC cell line (TUHR14TKB) and their doubling times decreased during passage. They also reported that the proliferation and the cell migration property of the cell line decreased when FABP7 was overexpressed. They concluded that the difference in FABP7 function between RCC cell lines suggests that FABP7 affects cell proliferation depending on cell phenotype [25].

Although *FABP6* mRNA was not highly differentially expressed in our transcriptome analysis, it does have high differential expression in ccRCC tissues compared to normal tissue according to TCGA database (Table 3) [26]. The expression level of *FABP6* mRNA is reported to be comparable to that of *FABP7* mRNA, while there are no reports showing prognostic significance of FABP6 in ccRCC. Ohmachi et al. reported that the expression of FABP6 is higher in primary colorectal cancers than in normal epithelium, but is decreased in lymph node metastases. They supposed that FABP6 may play an important role in early carcinogenesis [27]. Our study showed that FABP6 can complement FABP7 in a cell type-dependent manner and indicates that a knockdown of *FABP7* and/or *FABP6* can reduce viability and invasive potential of ccRCC cells. Specific inhibition of fatty acid binding proteins may be a novel strategy against ccRCC.

**Table 3:** mRNA expression level of FABP family in ccRCC

Gene	LSMean (Tumor)	LSMean (Normal Kidney)	Fold-Change (RCC/NK)
FABP1	605,588	14,528,517	0.04
FABP2	6465	37,070	0.17
FABP3	13,401,684	19,556,421	0.69
FABP4	1,330,864	2,052,261	0.65
FABP5	938,428	236,739	3.96
FABP6	4,804,806	65,430	73.43
FABP7	56,445,770	809,368	69.74

Cancer Genome Atlas Research Network

Comprehensive molecular characterization of clear cell renal cell carcinoma

Nature. 2013 Jul 4;499(7456):43–9

## Conclusions

Functional suppression of FABP7 significantly reduced cell viability and invasive potential in a ccRCC cell line. FABP7 may play a role in progression in some metastatic ccRCCs. The suppressed function may be compensated by another FABP family member.
